# Ridinilazole, a narrow spectrum antibiotic for treatment of *Clostridioides difficile* infection, enhances preservation of microbiota-dependent bile acids

**DOI:** 10.1152/ajpgi.00046.2020

**Published:** 2020-06-29

**Authors:** Xi Qian, Karin Yanagi, Anne V. Kane, Nicholas Alden, Ming Lei, David R. Snydman, Richard J. Vickers, Kyongbum Lee, Cheleste M. Thorpe

**Affiliations:** ^1^Tufts Clinical and Translational Science Institute, Tufts University, Boston, Massachusetts; ^2^Department of Chemical and Biological Engineering, Tufts University, Medford, Massachusetts; ^3^Tufts Medical Center, Boston, Massachusetts; ^4^Tufts University School of Medicine, Boston, Massachusetts; ^5^Summit Therapeutics, Plc, Abingdon, United Kingdom

**Keywords:** antibiotic therapy, bile acid, *Clostridioides difficile* infection, longitudinal outcome

## Abstract

Antibiotic treatment is a standard therapy for *Clostridioides difficile* infection, but dysbiosis of the gut microbiota due to antibiotic exposure is also a major risk factor for the disease. Following an initial episode of *C. difficile* infection, a relentless cycle of recurrence can occur, where persistent treatment-related dysbiosis predisposes the patient to subsequent relapse. This study uses a longitudinal study design to compare the effects of a narrow-spectrum (ridinilazole) or broad-spectrum antibiotic (vancomycin) on intestinal bile acid profiles and their associations with gut bacteria over the course of *C. difficile* infection treatment. At the end of treatment (*day 10*), subjects receiving vancomycin showed a nearly 100-fold increase in the ratio of conjugated to secondary bile acids in their stool compared with baseline, whereas subjects receiving ridinilazole maintained this ratio near baseline levels. Correlation analysis detected significant positive associations between secondary bile acids and several Bacteroidales and Clostridiales families. These families were depleted in the vancomycin group but preserved at near-baseline abundance in the ridinilazole group. Enterobacteriaceae, which expanded to a greater extent in the vancomycin group, correlated negatively and positively with secondary and conjugated primary bile acids, respectively. Bile acid ratios at the end of treatment were significantly different between those who recurred and those who did not. These results indicate that a narrow-spectrum antibiotic maintains an intestinal bile acid profile associated with a lowered risk of recurrence.

**NEW & NOTEWORTHY** This is the first study to demonstrate in humans the relationships between *Clostridioides difficile* antibiotic treatment choice and bile acid metabolism both during therapy and after treatment cessation. The results show a microbiota- and metabolome-preserving property of a novel narrow-spectrum agent that correlates with the agent’s favorable sustained clinical response rates compared with broad-spectrum antibiotic treatment.

## Graphical abstract

**Figure:**
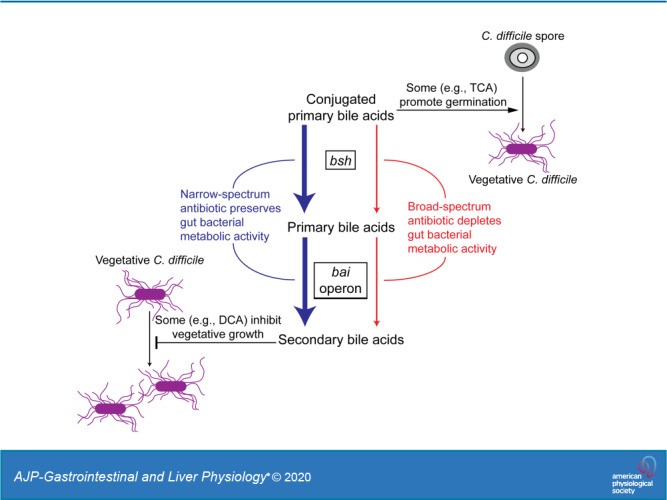

## INTRODUCTION

*Clostridioides difficile* is a spore-forming organism that can colonize the human intestine. In susceptible individuals, *C. difficile* infection (CDI) results in significant morbidity and mortality. On average, recurrence of CDI following antibiotic treatment is seen in ∼20% of cases (26), with increasing rates of recurrence after the first recurrence (reviewed in Ref. 38). Disruption of the intestinal microbiota by antibiotic treatment is a major risk factor for CDI. An increasing body of evidence suggests that the intestinal bile acid profile plays an important role in both development of disease and resistance to recurrence (35, 53). Commensal bacteria in the intestine metabolize conjugated bile acids by the activity of two sets of enzymes. Bile salt hydrolases (BSHs) remove the conjugated taurine or glycine to generate unconjugated bile acids. Unconjugated primary bile acids can be further metabolized by the 7α-dehydroxylation pathway to produce secondary bile acids. In vitro, the conjugated and unconjugated primary bile acids taurocholate (TCA) and cholate (CA), respectively, promote *C. difficile* spore germination, whereas secondary bile acids such as lithocholate (LCA) and deoxycholate (DCA) generally inhibit vegetative growth.

However, sporulation and growth are complex processes (56), and different strains of *C. difficile* exhibit varying responses to bile acids (39). For example, a recent study using *C. difficile* 630Δ*erm* showed that both DCA and chenodeoxycholate, a primary bile salt, promote biofilm formation, an effect that is potentiated by fermentable sugars (14). Furthermore, the study reports that cells in DCA-induced biofilm show markedly lower sensitivity to vancomycin and metronidazole compared with planktonic cells, whereas DCA exposure enhances the sensitivity of planktonic cells to the antibiotics. Taken together, these studies indicate that bile acids can directly affect *C. difficile* germination and growth while underlining the complexity of these effects, which vary depending on bile acid and growth environment. The in vitro findings are consistent with studies in mice, which also showed that resistance to *C. difficile* germination and growth is strongly associated with the presence of specific secondary bile acids (41). Moreover, the murine studies found that antibiotic treatment leads to a loss in secondary bile acids (41).

In humans, several studies have linked differences in bile acid levels to specific clinical states. These studies generally report higher primary bile acids in recurrent *C. difficile* patients and higher secondary bile acids in healthy subjects or patients with recurrent CDI who have undergone successful fecal microbiota transplantation (FMT) (2, 3, 8, 34, 51, 52). Normalization of intestinal microbial community structure in patients undergoing FMT occurs concomitant with restoration of secondary bile acid levels. In the colon, formation of secondary bile acids such as LCA and DCA depends on expression of genes in the bile acid inducible (*bai*) operon encoding key enzymes of the 7α-dehydroxylation pathway (19). Unlike genes encoding BSH, which are widely distributed among gut bacteria of different phyla, the *bai* operon appears to be confined to a relatively small number of species, one of which is *Clostridium scindens*. An analysis of microbiome data from CDI-diagnosed and *C. difficile* carrier patients undergoing allogeneic hematopoietic stem cell transplantation found a strong association between the presence of *C. scindens* and *C. difficile* colonization resistance (9).

Whereas murine model and human FMT studies have shown strong associations between CDI recurrence and intestinal bile acid profiles, relatively little has been reported regarding the effects of different antibiotic treatments on bile acid levels in humans. Previously, bile acid levels in humans with CDI have been measured only at a single time point or before and after an intervention such as FMT. In this study, we characterize the trajectory of stool bile acid levels over time in human subjects diagnosed with CDI enrolled in a phase 2 clinical trial comparing the treatment efficacy of a broad-spectrum agent (vancomycin) against a narrow spectrum investigational agent (ridinilazole) (47). In the phase 2 trial, treatment with ridinilazole was associated with fewer recurrences. We have previously shown that vancomycin-treated subjects showed dramatic losses of Lachnospiraceae, Ruminococcaceae, and Bacteroidaceae in stool collected at the end of treatment (EOT), with concomitant expansion of species belonging to the phylum proteobacteria (43). In contrast, ridinilazole-treated subjects showed minimal disturbance in their gut microbiota compared with when the subjects enrolled in the trial (baseline). Based on these observations, we tested the hypothesis that secondary bile acid levels would be depleted in the stool of vancomycin-treated subjects compared with ridinilazole-treated subjects and that the depletion of bile acids would correlate with losses of specific gut bacteria.

## MATERIALS AND METHODS

### Materials.

Glycocholic acid (GCA) and glycodeoxycholic acid (GDCA) were purchased from Cayman Chemical (Ann Arbor, MI). Glycolithocholic acid (GLCA) was purchased from Isosciences (Ambler, PA). Unless otherwise noted, all other chemicals were purchased from Sigma-Aldrich (St. Louis, MO).

### Participants and sample collection.

Stool samples were collected at multiple time points as part of a recent phase 2, double-blind, randomized, controlled, noninferiority clinical trial, as described (43). Institutional review boards at each enrolling center provided ethics approval, and the study complied with the ethical principles expressed in the Declaration of Helsinki and followed all principles of good clinical practice. Written, informed consent was obtained from all participants. The Tufts Institutional Review Board determined that the present study using deidentified stools constituted nonhuman subject research. In the trial, 100 subjects were enrolled at 33 medical centers in the United States and Canada and randomized at a 1:1 allocation ratio to receive 10 days of either vancomycin or ridinilazole. Participants were recruited between June 26, 2014, and August 31, 2015. Stool samples were obtained at study entry (baseline), end of treatment [EOT; *day 10* (D10)], *day 25* (D25), and *day 40* postentry (D40) and when a recurrence was suspected. Of the 100 subjects enrolled in the clinical trial, 18 provided fewer than three stool samples for analysis and were eliminated from this study. Of the remaining 82 participants, there were 41 per treatment arm. Because any antibiotic therapy can alter the intestinal microbiota and thus introduce confounding effects, participants who received standard CDI treatments (metronidazole or vancomycin) up to 24 h before randomization and/or who were receiving nonCDI related antibiotic therapy at study enrollment were excluded from this analysis to capture the effects of ridinilazole or vancomycin alone (for consort diagram, see Supplemental Fig. S1; Supplemental Material for this article can found online at https://doi.org/10.6084/m9.figshare.11859225.v5). This reduced the study groups to 22 subjects per treatment arm. When new antibiotics for either CDI recurrence or a new infection were started, subsequent samples were censored from the analysis, unless otherwise noted. There were no significant differences in age, sex, body mass index (BMI), or use of proton pump inhibitors (PPIs), nonsteroidal anti-inflammatory drugs (NSAIDS), opioids, or probiotics between treatment groups. Control stool samples were obtained from volunteers enrolled in a separate study (44), because the phase 2-only enrolled subjects diagnosed with CDI. The mean ages of the treatment groups and healthy controls are not significantly different. Importantly, the stool samples from all subjects (CDI subjects and healthy control) were handled, stored, processed, and analyzed for microbiota composition and metabolite contents using identical methods. This eliminated potentially confounding influences that could be present due to methodological differences between the two studies.

### Extraction of metabolites from stool samples.

Frozen stool samples stored at −80°C were lyophilized overnight in 2.0-mL screw-capped tubes (Sarstedt). After recording the dry weight, 0.5 g of 0.1-mm diameter zirconia/silica beads (BioSpec Products) and 1.5 mL of an ice-cold chloroform-methanol (2:1, vol/vol) solution were added to each sample tube. The tubes were then held on a bead-beater (Vortex Genie 2 with MoBio Vortex adapter) for 5 min at 4°C. The debris was pelleted by centrifugation at 16,000 *g* for 10 min. The supernatant was carefully removed and filtered over a 70-μm cell strainer (Thermo Fisher Scientific) into a fresh sample tube. After the addition of ice-cold HPLC-grade water at 60% of the filtrate’s volume, the sample tube was vortexed for 20 s and centrifuged at 16,000 *g* and 4°C for 10 min. The polar layer was then carefully removed using a syringe and dried in a Savant Speed-Vac (Thermo Fisher Scientific) for 5 h. The dried extracts were stored at −80°C before metabolite analysis. Of note, all stool samples underwent a freeze-thaw cycle before extraction; this did not affect bile acid levels.

### Targeted analysis of bile acids.

The dried samples were reconstituted in 100 μL of methanol/water (1:1, vol/vol). Bile acids were quantified using targeted liquid chromatography-mass spectrometry (LC-MS) experiments performed on a triple-quadrupole mass analyzer (QQQ 6410A; Agilent, Santa Clara, CA). A previously described multiple reaction monitoring (MRM) method (33) was modified to improve separation between bile acids of similar mass and chemical structure. Chromatographic separation was achieved on a C18 column (Kinetex 5 μm EVO C18 100A, 150 × 2.1 mm; Phenomenex) using a gradient method with two mobile phases (Supplemental Table S1, https://doi.org/10.6084/m9.figshare.12217979.v1). *Solvent A* was methanol-water (1:1, vol/vol) with 10 mM ammonium acetate and 0.1% (wt/vol) ammonium hydroxide (pH 9). *Solvent B* was methanol with the same concentrations of ammonium acetate and ammonium hydroxide as *solvent A* (pH 9). The injection volume was 10 μL, and column oven temperature was set to 50°C. The mass spectrometer was operated in negative ionization (ESI−) mode. The optimized MRM transitions and retention times of chemical standards are shown in Supplemental Table S2, https://doi.org/10.6084/m9.figshare.12217979.v1). The MRM scan data were acquired using MassHunter (version B.05.00; Agilent) and imported into Skyline (27) for peak identification and integration of areas under the curve (AUCs). Peaks in the ion chromatograms were identified based on retention time (RT) and MRM transition. For quantification of bile acids, concentrations were calculated from the AUCs using standard curves generated from high-purity standards. The bile acid concentrations were normalized by the corresponding sample dry weight.

### Estimation of BSH gene abundance.

The PICRUSt2 software package (https://github.com/picrust/picrust2) was used to estimate the distribution of BSH gene abundances in the stool samples. Metagenome functional content was predicted from 16S rRNA survey data collected in previous studies (43, 44) on the same set of samples analyzed for metabolites in this study. Standard procedures were followed to process the 16S rRNA data using QIIME (10) and generate an operational taxonomic unit (OTU) table as input for PICRUSt2. Briefly, PICRUSt2 uses an evolutionary model and complete sequenced genomes as a reference tree of life to compute an estimate for gene family copy numbers of bacterial types or OTUs. The abundance of each OTU is divided by its predicted 16S rRNA copy number and then multiplied by the copy numbers of gene families. The result is an estimate for each OUT’s contribution to a sample’s overall gene content or metagenome.

### Untargeted analysis of stool metabolites.

The untargeted LC-MS experiments were performed on the same extracted samples as the targeted experiments on bile acids. The samples were analyzed for global metabolite profiles using information-dependent acquisition (IDA) experiments performed on a triple-quadrupole time-of-flight (TOF) instrument (5600+; AB Sciex) coupled to a binary pump HPLC system (1260 Infinity; Agilent). Each sample was analyzed four times using a combination of positive and negative ionization modes and two different LC methods (Supplemental Methods, https://doi.org/10.6084/m9.figshare.12217979.v1) to obtain broad coverage of metabolites having varying polarities and isoelectric points. Raw data were preprocessed using XCMS (13) to extract and align peaks. The peaks were then processed using CAMERA (24) to identify and remove predicted isotopes, adducts, and in-source fragments. The retained peaks were organized into a feature table, with each feature specified by accurate mass-to-charge ratio (*m/z*), retention time (RT), and responses representing the AUC for the extracted ion chromatogram of the feature. The AUC of a feature was normalized by the corresponding sample dry weight. In the case that a precursor ion detected by the IDA’s survey scan triggered an MS/MS scan, the corresponding MS/MS spectrum was extracted and searched against the spectral libraries of HMDB (54) and NIST17 (21). The MS/MS spectrum of each feature was also analyzed using in silico fragmentation tools MetFrag (55) and CFM-ID (4). These analyses identified several annotations for many of the features. To determine the most likely identities for these features in the context of human intestinal metabolism, we applied an automated annotation procedure (“BioCAn”) that combines the outputs from the database searches and fragmentation analyses with a metabolic model for the biological system of interest (1). Briefly, BioCAn maps each unique mass in the feature table onto a metabolic network representing the enzymatic reactions possible in the system of interest and evaluates the likelihood that a correct mapping between a detected mass and a metabolite in the network has occurred based on two factors: how many other metabolites in the neighborhood (defined by reaction distance) of the metabolite in question also map to a detected mass and the relative confidence in the presence of that compound and its neighbors based on results from MS/MS matching to spectral databases and in silico tools (Supplemental Methods).

The metabolic network for BioCAn was assembled from the predicted metagenomes obtained using PICRUSt2. A separate network was assembled for annotation of ridinilazole and vancomycin samples to account for the differences in microbiomes between the two treatment groups. The gene functions predicted by PICRUSt2 for ridinilazole and vancomycin samples were tabulated by their KEGG Orthology identifiers (K numbers) and estimated function counts (22). After nonenzymatic functions were eliminated, the K numbers were further filtered to remove functions that have very low counts (median count <10 across all ridinilazole or vancomycin samples) and are thus unlikely to play a quantitative role in microbiota metabolism. The remaining enzymatic functions were then linked to KEGG reaction identifiers (*r* numbers) and their primary substrate-product pairs as defined by KEGG’s RCLASS data (46). The substrate-product pairs were used to draw the network graphs for annotation, where the nodes and edges correspond to metabolites and reactions, respectively.

### Correlation analysis.

To determine whether there are significant associations between specific bile acids and bacterial groups, rank correlation coefficients (Spearman’s rho) were calculated between OTU counts (relative abundance) and bile acid concentrations. To lessen the chance of detecting spurious correlations, OTUs were excluded when detected in <5% of the samples, although this may result in missing true correlations with low-abundance organisms. The remaining 1,488 OTUs were grouped by family. In a handful of cases, OTU picking did not resolve the organism at the family level. In these cases, the OTUs were grouped by order. This resulted in a reduced OTU table of 35 families and three orders. Correlations (rho values) were calculated for each bile acid and family/order across samples from ridinilazole-treated subjects, vancomycin-treated subjects, antibiotic-treated CDI subjects (ABX), and all subjects (ALL), including healthy controls. The *P* values were corrected for false discovery rate (FDR) using the Benjamini-Hochberg (B-H) method (6). A corrected *P* value of <0.05 was considered statistically significant.

### Statistical analysis.

Within-group differences in bile acid levels between time points (e.g., ridinilazole baseline vs. ridinilazole EOT) were tested by using a Wilcoxon signed-rank test, and the differences between groups at the same time point (e.g., ridinilazole EOT vs. vancomycin EOT) were analyzed by using a Wilcoxon rank-sum test. A *P* value of <0.05 was considered statistically significant. To test whether the changes over time in bile acid composition differed between treatment groups, the relative abundance of each bile acid group (primary, secondary, conjugated primary, or conjugated secondary) at each time point was summarized as an average percentage of total bile acids and analyzed using a linear mixed-effects model (MIXED procedure, SAS 9.4). The model included treatment, time, and their interactions as fixed effects. Bayesian information criterion was used to determine the best-fitting covariance structure, which was first-order autoregressive. We used random forest (Supplemental Methods) to build a classification model for the purpose of identifying individual bile acids and bile acid ratios that can best predict whether the sample was obtained from a subject treated with ridinilazole or vancomycin. For the untargeted metabolites analysis, significance of differences between ridinilazole and vancomycin treatment groups at EOT was determined using Student’s *t* test. The *P* values from multiple comparisons were corrected for FDR using the B-H method (6).

## RESULTS

### Ridinilazole treatment preserves secondary bile acids.

We compared the effects of a broad-spectrum antibiotic, vancomycin, which is commonly used to treat CDI, against the effects of a selectively targeting antibiotic, ridinilazole (47), on the bile acids listed in Table 1. The concentrations of these bile acids in stool samples from vancomycin- or ridinilazole-treated subjects (in μmol/g stool dry wet), as well as in stool samples from a cohort of healthy subjects, are shown in Supplemental Figure S2 (https://doi.org/10.6084/m9.figshare.11859228). Bile acids and their groupings that were quantified in this study are shown in Table 1.

**Table 1.** Bile acids measured in this study

Group (Name)	Abbreviation
Primary	
Cholate	CA
Chenodeoxycholate	CDCA
Conjugated primary	
Glycocholate	GCA
Taurocholate	TCA
Glycochenodeoxycholate	GCDCA
Taurochenodeoxycholate	TCDCA
Secondary	
Lithocholate	LCA
Deoxycholate	DCA
Ursodeoxycholate	UDCA
Hyodeoxycholate	HDCA
Conjugated Secondary	
Glycolithocholate	GLCA
Taurolithocholate	TLCA
Glycodeoxycholate	GDCA
Taurodeoxycholate	TDCA

**Fig. 1.**
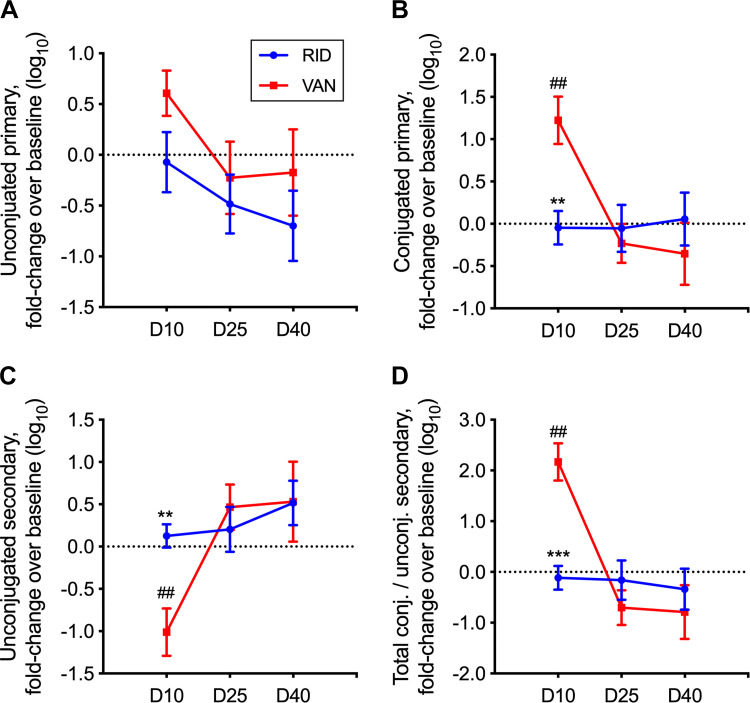
Fold change of bile acids over time. Fold changes in concentrations of unconjugated primary bile acids (*A*), conjugated primary bile acids (*B*), and unconjugated secondary bile acids (*C*) and ratio of total conjugated (conjugated primary and conjugated secondary bile acids) to unconjugated secondary bile acids (*D*) were calculated for each subject relative to the subject’s baseline, with concentrations measured in μmol/g dry stool weight. Data shown are means ± SE. See Table 1 for bile acid groupings. The sample numbers for fold change in the vancomycin (VAN) group were 19 on *day 10* (D10), 14 on *day 25* (D25), and 10 on *day 40* (D40); the numbers in the ridinilazole (RID) group were 21 on *day 10* (D10), 18 on *day 25* (D25), and 17 on *day 40* (D40). ##Significant differences from baseline within each treatment group by Wilcoxon signed-rank test (*P* < 0.001); **P < 0.001 and ***P < 0.0001, significant differences between treatment groups by Wilcoxon rank-sum test.

The total concentration of unconjugated primary bile acids (referred to hereafter simply as primary bile acids) trended similarly for both ridinilazole and vancomycin subjects (Fig. 1*A*). A significant difference was observed for total conjugated primary bile acids. Subjects receiving ridinilazole showed no significant change from their individual baselines in conjugated primary bile acids throughout the study period (Fig. 1*B*). In contrast, subjects receiving vancomycin showed an order-of-magnitude increase in conjugated primary bile acids at EOT compared with baseline (*P* < 0.001). A significant difference between ridinilazole and vancomycin groups was also observed for secondary bile acids, which result from metabolism of unconjugated primary bile acids via a bacterially encoded 7α-dehydroxylation pathway. Whereas ridinilazole subjects exhibited a stable secondary bile acid profile relative to baseline, vancomycin subjects showed an ∼10-fold decrease (*P* < 0.01) in these bile acids at EOT before they returned to baseline on D25 and D40 (Fig. 1*C*). The ratio of total conjugated (primary and secondary) to secondary bile acids was examined. This ratio is the most important classifier of treatment group at EOT, as determined by a random forest model (Supplemental Fig. S3, https://figshare.com/articles/Figure_S3/11859246), and is also a measure of both bacterial BSH activity and 7α-dehydroxylation pathway enzymatic activity on the stool bile acid pool. At baseline, the ratio was not significantly different between the ridinilazole and vancomycin groups (1.5 ± 0.7 and 4.6 ± 4.0, respectively). However, there was an ∼100-fold increase (*P* < 0.0001) in this ratio at EOT relative to baseline for the vancomycin group, whereas the ratio was maintained near baseline for the ridinilazole group (Fig. 1*D*).

### Ridinilazole treatment maintains bile acid composition.

**Fig. 2.**
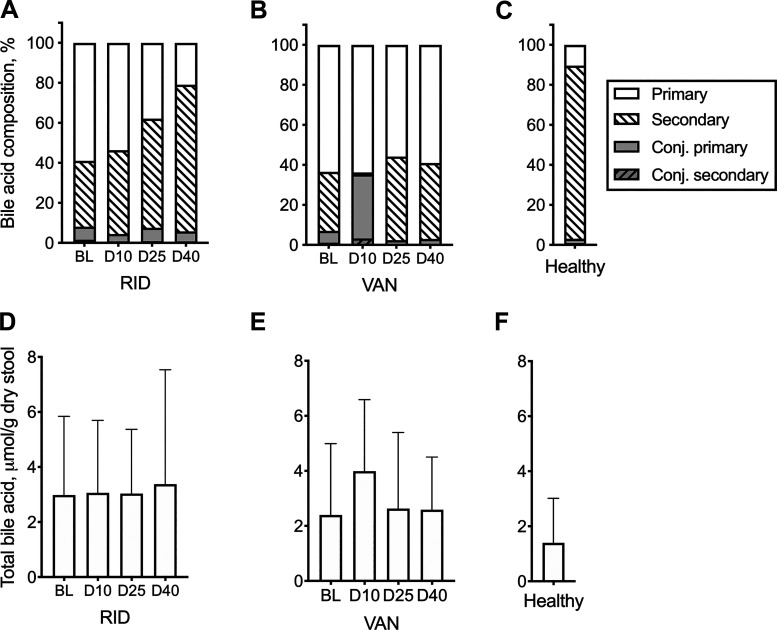
Bile acid (BA) composition in treated subjects and healthy controls. BA composition was calculated for ridinilazole (RID; *A*), vancomycin (VAN; *B*), and healthy subjects (*C*) as %total/g dry wt of stool. Corresponding total BA concentrations are shown in *D*–*F*, respectively. See Table 1 for BA groupings. Differences in changes across time and treatment were assessed using a linear mixed-effect model (SAS 9.4). Statistically significant outcomes were relative abundances of secondary and conjugated primary BA. BL, baseline; D10, *day 10*; D25, *day 25*; D40, *day 40*.

We next compared the effect of ridinilazole or vancomycin on the relative abundances of bile acids at different time points. This analysis was undertaken because within the stool matrix, organisms encounter complex mixtures of bile acids. In vitro, combinations of bile acids have been shown to affect *C. difficile* germination differently than a single bile acid when given alone in the same concentration (39). Figure 2 shows the total bile acid concentration (Fig. 2, *A*–*C*) and relative abundance of each bile acid category (primary, secondary, conjugated primary, or conjugated secondary) as an average percentage of total bile acids (Fig. 2, *D*–*F*). Differences in changes across time and treatment were assessed using a linear mixed-effects procedure. No significant differences between time points or treatment groups were found for total bile acid. At baseline, vancomycin- and ridinilazole-treated subjects had similar bile acid compositions, with unconjugated (primary and secondary) bile acids accounting for >90% of the total in both groups. At EOT (D10), vancomycin-treated subjects had a significant increase in the average proportion of conjugated primary bile acids (35%, *P* < 0.0001) and a significant decrease in the average proportion of unconjugated secondary bile acids (<1%, *P* < 0.001) compared with ridinilazole-treated subjects. On D40, both ridinilazole- and vancomycin-treated subjects had similarly high average proportions of unconjugated bile acids (97% and 94% for vancomycin and ridinilazole, respectively). There was no longer a statistically significant difference in the average proportion of conjugated primary bile acids between the two groups. However, the ridinilazole group still had a significantly greater (*P* < 0.01) average proportion of secondary bile acids (73%) compared with the vancomycin group (38%), and there was a statistically significant difference in primary bile acids (*P* < 0.01). The bile acid composition of the ridinilazole group more closely approached that of a cohort of healthy subjects (disease free, no antibiotic exposure).

**Fig. 3.**
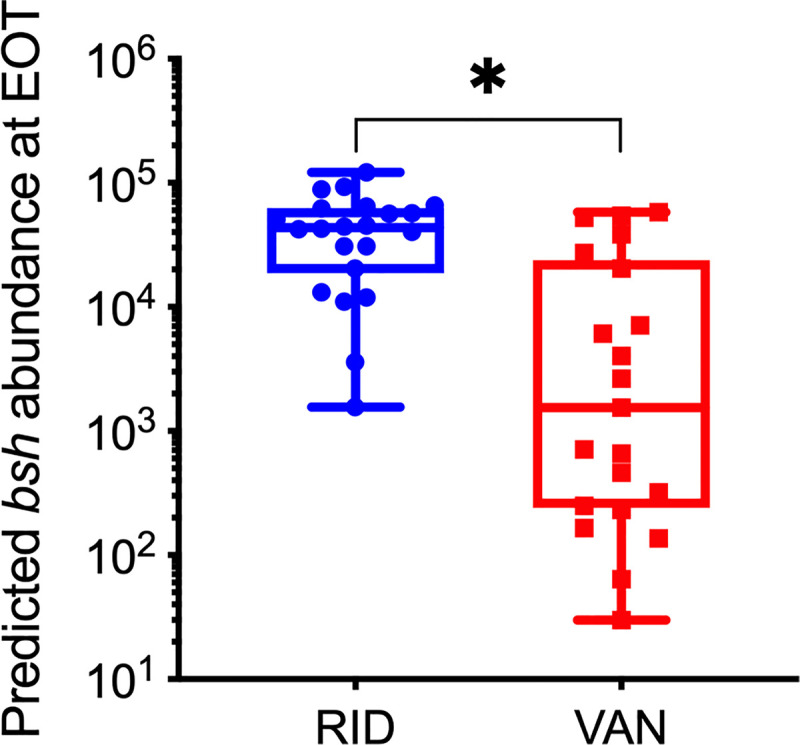
Bile salt hydrolase (BSH) gene abundance at end of treatment (EOT). Gene abundance was estimated from taxonomic abundance data [operational taxonomic unit (OTU) table] using PICRUSt2. Dot plot shows estimated *bsh* abundance for ridinilazole- (RID; blue circles) and vancomycin-treated (VAN; red squares) subjects. Box plot shows medians and interquartile ranges. *P < 0.0001, statistically significant difference between treatment arms by the Wilcoxon rank-sum test.

To determine whether differences in bile acid levels and composition could reflect differences in BSH activity, the relative abundance of BSH genes was estimated using PICRUSt2. The predicted BSH gene abundance in vancomycin-treated subjects at EOT was more than threefold lower than in ridinilazole-treated subjects (*P* < 0.0001; Fig. 3).

### Primary and secondary bile acids are associated with distinct families of gut bacteria.

**Fig. 4.**
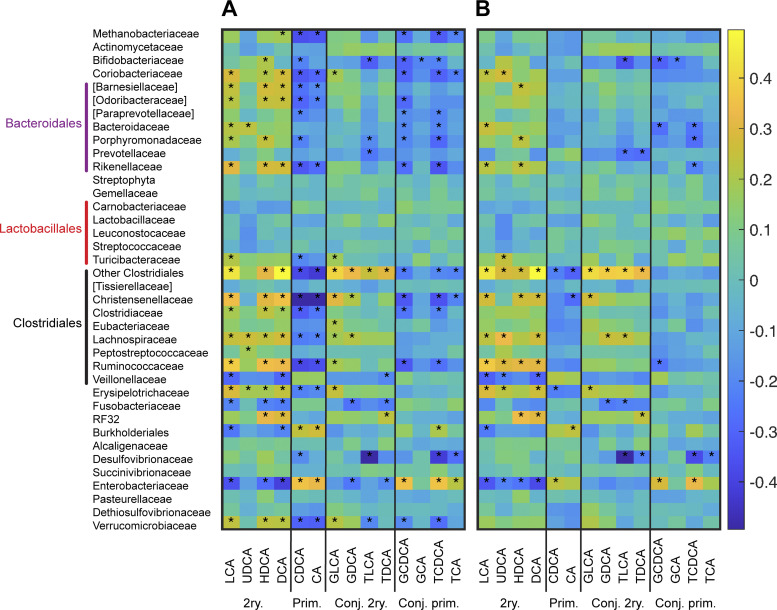
Correlations of microbiota with bile acids. *A*: overall bile acid-microbiota correlations for all (treated + control) subjects, grouped by bacterial family. *B*: aile acid-microbiota family correlations in treated subjects, grouped by family. Heatmap shows Spearman’s rank correlation values. Operational taxonomic units (OTUs) were excluded from the correlation analysis when detected in <5% of the samples. See Table 1 for bile acid abbreviations. *P < 0.05, significant correlation after false discovery rate (FDR) correction. Included in this analysis are 2 samples from ridinilazole (RID) patients [1 each on *day 25* (D25) and *day 40* (D40)], and 4 samples from vancomycin (VAN) subjects (1 on D25 and 3 on D40) that were obtained after these subjects had begun new antibiotics as well as 10 samples (3 RID, 7 VAN) taken at the time of suspected recurrence. 2ry., secondary bile acid; CA, cholate; CDCA, chenodeoxycholic acid; Conj. 2ry., conjugated secondary bile acid; Conj. prim., conjugated primary bile acid; DCA, deoxycholic acid; GCA, glycocholic acid; GCDCA, glycochenodeoxycholic acid; GLCA, glycolithocholic acid; HDCA, hyodeoxycholic acid; LCA, lithocholic acid; Prim., primary bile acid; TCDCA, taurochenodeoxycholic acid; TLCA, tauroursodeoxycholic acid; UDCA, ursodeoxycholic acid.

We next investigated whether the variations in bile acids could be associated with enrichment or depletion of specific groups of gut bacteria. Of note, as this analysis was focused on taxa-bile acid correlations, we included two samples from ridinilazole patients (1 each on D25 and D40) and four samples from vancomycin subjects (1 on D25 and 3 on D40) that were obtained after these subjects had begun new antibiotics as well as 10 samples that were taken at the time of suspected recurrence. Across all subjects (ridinilazole, vancomycin, and healthy subjects), significant positive correlations were detected between secondary bile acids and several families in Bacteroidales (Bacteroidaceae and Rikenellaceae) and Clostridiales (Christensenellaceae, Clostridiaceae, Lachnospiraceae, and Ruminococcaceae; Supplemental Fig. S4, https://doi.org/10.6084/m9.figshare.12214691.v1) and the family Coriobacteriaceae (Fig. 4*A*). These bacterial families also correlate negatively with primary bile acids, suggesting that they could contribute to the enzymatic conversion of primary to secondary bile acids. An opposite trend was found for Enterobacteriaceae (Supplemental Fig. S4); this family of Proteobacteria correlates positively with conjugated primary bile acids and negatively with secondary bile acids. Bifidobacteriaceae show the strongest negative correlation with conjugated bile acids, consistent with genomic data showing that many species in this family harbor BSH (31).

These trends largely hold for ridinilazole and vancomycin samples analyzed together (healthy controls excluded), although there are fewer significant correlations (Fig. 4*B*). In contrast, samples from healthy subjects alone lack any significant correlations (data not shown). The number of significant correlations is further reduced when the ridinilazole and vancomycin samples are analyzed separately (Supplemental Fig. S5, https://doi.org/10.6084/m9.figshare.11859252.v2). Between the two antibiotics, fewer correlations were detected for the vancomycin group. Whereas ridinilazole samples still show significant correlations involving Enterobacteriaceae (negative correlation) and several families in Clostridiales (positive correlations), these correlations are no longer detected in the vancomycin samples. Overall, this suggests that detection of correlations is primarily driven by the magnitude of OTU and bile acids variations resulting from CDI and/or antibiotic treatment.

### Untargeted analysis corroborates differences in bile acid metabolism between ridinilazole- and vancomycin-treated subjects.

**Fig. 5.**
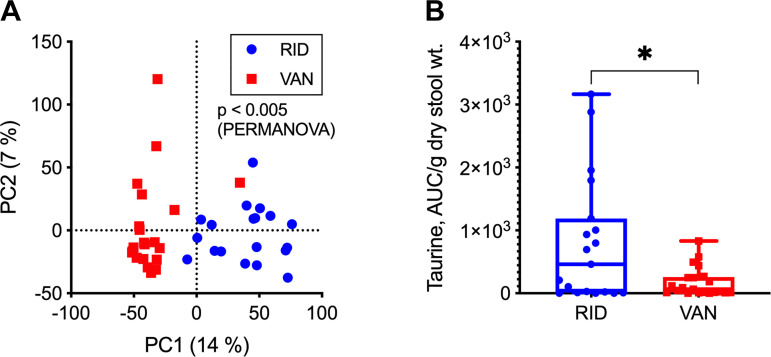
Untargeted analysis of end-of-treatment (EOT) samples. *A*: scatter plot of the first 2 principal component (PC) scores of liquid chromatography-mass spectrometry (LC-MS) features. Numbers in parentheses show %variation explained by the corresponding PC. The LC-MS data were autoscaled before principal component analysis. Each blue circle or red square represents, respectively, a ridinilazole (RID)- or vancomycin (VAN)-treated subject. The *P* value (<0.001) comparing the between-group and within-group variances of the 2 treatment groups was calculated using PERMANOVA on autoscaled LC-MS data, with Euclidean distance as the dissimilarity metric. *B*: dot plot shows taurine levels [reported as extracted ion chromatogram area under the curve (AUC) normalized to dry sample weight] in stool from RID- (blue circles) and VAN-treated (red squares) subjects. Box plot shows median and interquartile ranges. *P < 0.05, a statistically significant difference between treatment groups by the Wilcoxon rank-sum test after false discovery rate correction.

To determine whether there were other major metabolic differences between the ridinilazole and vancomycin groups, we more broadly characterized the subjects’ fecal metabolome using untargeted LC-MS experiments. We focused this analysis on EOT, as this time point showed the greatest differences in both bile acids and predicted metagenomes. A scatterplot of the first two principal component scores (PC1 and PC2) derived from all monoisotopic LC-MS features shows nearly complete separation of ridinilazole and vancomycin samples along PC1 (Fig. 5*A*).

The PCA result was corroborated by a multivariate test (PERMANOVA, *P* < 0.001) as well as statistical tests on individual features. After FDR correction, we found 4,066 features that were present at significantly different levels in ridinilazole and vancomycin groups. More than 83% of these features were elevated in the ridinilazole group. Using our automated data annotation procedure, we putatively identified 142 significant metabolites that could be mapped to at least one functional category, i.e., KEGG pathway map, predicted by PICRUSt2. Approximately 87% of the putatively identified metabolites were elevated in the ridinilazole group, similar to the overall feature table.

Pathway analysis using MetaboAnalyst (11) found four pathways that are overrepresented in the putatively identified set of metabolites detected at significantly different levels. Half of these pathways were related to bile acid metabolism, specifically taurine metabolism (*P* < 0.01) and primary bile acid synthesis (*P* < 0.05). The bile acid trends from the untargeted analysis are in good agreement with the results from targeted analysis (Supplemental Fig. S6, https://doi.org/10.6084/m9.figshare.12217949.v1). We also detected a significantly higher level of free taurine in the ridinilazole group, consistent with the increase in taurine-conjugated bile salts in the vancomycin group (Fig. 5*B*).

### Secondary bile acids correlate with recurrence.

**Fig. 6.**
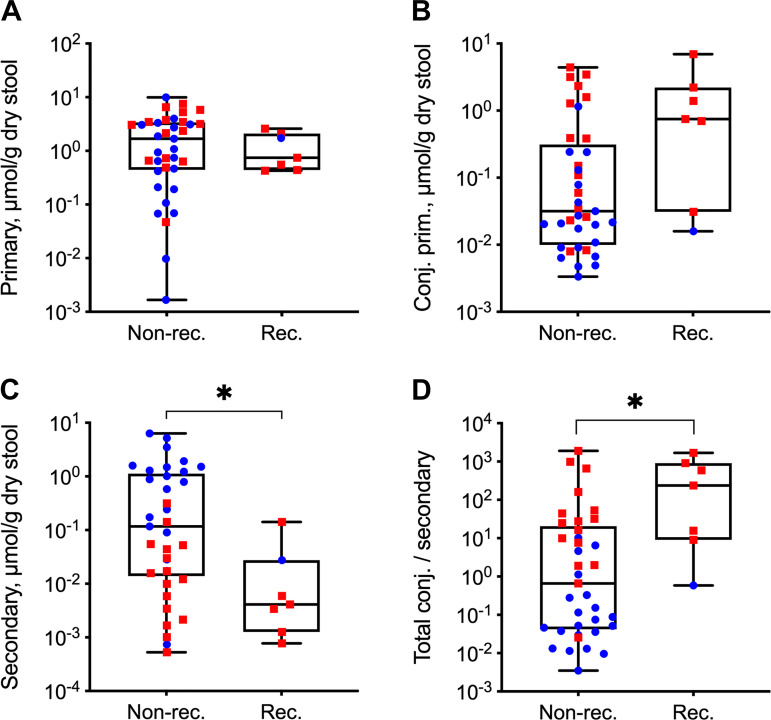
Bile acid levels at end of treatment (EOT) in confirmed recurrence vs. nonrecurrence subjects. Dot plot shows concentrations of bile acids or bile acid ratios at EOT for subjects who had (*n* = 7 subjects) or did not have (*n* = 35 subjects) a subsequently confirmed recurrence of *C. difficile* infection (CDI) during the study. See Table 1 for bile acid groupings. Box plot shows median and interquartile ranges. Data shown are log_10_-transformed concentrations (μmol/g dry stool weight) of primary bile acids (*A*), conjugated primary bile acids (*B*), and secondary bile acids (*C*) and ratio of total conjugated (conjugated primary and conjugated secondary) to secondary bile acids (*D*). *P < 0.05, statistically significant difference between recurrence and nonrecurrence subjects by Wilcoxon rank-sum test.

Because of the limited number (only 1) of ridinilazole-treated subjects with confirmed CDI recurrence, a statistical analysis on the differences between treatment groups in the subset of subjects with recurrence could not be performed. To explore whether bile acid profiles correlated with recurrence regardless of the treatment, we compared samples from all subsequently recurrent (*n* = 7 subjects) and nonrecurrent subjects (*n* = 35 subjects) in the entire study cohort at EOT (Fig. 6). There was no association between recurrence and any of the individual primary bile acids (data not shown). However, subjects who subsequently suffered from a recurrence of CDI had a significantly lower level of secondary bile acids. Additionally, subjects with recurrence had a significantly higher ratio of total conjugated to secondary bile acids. The concentrations of conjugated primary bile acids were lower in subjects who did not recur compared with those who did, but this difference was not statistically significant. Primary bile acid and taurine concentrations were not different.

## DISCUSSION

### Comparison of bile acid profiles between ridinilazole- and vancomycin-treated subjects.

This is the first study to demonstrate in humans the relationships between CDI antibiotic treatment choice and bile acid metabolism both during therapy and after treatment cessation. We discovered that ridinilazole and vancomycin had distinct impacts on stool bile acid profiles in CDI subjects at EOT and posttherapy (Figs. 1 and 2). Vancomycin treatment resulted in a significant increase of primary and conjugated bile acids, which was accompanied by a significant decrease in secondary bile acids at EOT compared with baseline. Our results on vancomycin-treated subjects with CDI resemble bile acid profiles associated with vancomycin-induced dysbiosis described in previously published human studies on non-CDI subjects with inflammatory bowel disease and primary sclerosing cholangitis (45), obesity (5, 30, 48), or prediabetes (5). Similar alterations in stool bile acid profile following vancomycin administration have been observed in murine studies (25, 40). In striking contrast to vancomycin, ridinilazole preserved the levels of primary bile acids, conjugated bile acids, and secondary bile acids in the stool samples from CDI subjects at EOT relative to baseline. Moreover, the bile acid composition of the ridinilazole-treated subjects trended toward that of a cohort of healthy subjects 40 days after diagnosis of CDI as compared with the vancomycin group.

The apparent differential effects of ridinilazole and vancomycin on bile acid homeostasis is likely due to their distinct impacts on intestinal microbiota, as metabolism of conjugated primary bile acids to secondary bile acids in the distal small intestine and colon depends on bacterial enzymes (49). Our previous study in this cohort showed that vancomycin treatment resulted in microbiota-wide changes at EOT with significant reductions in relative abundances of Firmicutes, Bacteroidetes, and Actinobacteria (43). The same study showed that ridinilazole had a markedly narrower impact on the microbiota, which could explain the improved preservation of bile acid-metabolizing capability. We here observed higher predicted BSH gene abundance (Fig. 4) and higher taurine levels (Fig. 6). The higher taurine levels are consistent with higher bile salt hydrolase activity, as this is an end product of that reaction. Finally, we observed a reduction in stool bacterial biomass after vancomycin treatment in our previous study (43), which also likely plays a role in decreased metabolic capacity, not just the relative abundance of organisms with bile acid-metabolizing ability. Together, these findings suggest that preserved bile acid homeostasis by ridinilazole reflects its narrow spectrum.

### Associations between bile acids and microbial taxa in humans.

Whereas there have been a number of murine studies on the effects of antibiotic-induced dysbiosis on bile acids, only a handful have examined vancomycin, and these used vancomycin in conjunction with other broad-spectrum antibiotics, e.g., cefoperazone (42). We do not discuss these murine studies to any great extent, because there are qualitative differences in the bile acid profiles of humans and mice. Some bile acids that are quantitatively important in mice; e.g., muricholic acids are absent in humans. Furthermore, nearly all (∼95%) of the bile salts in mice are conjugated to taurine rather than glycine. In contrast, a majority (∼75%) of human bile salts are conjugated to glycine.

Previous studies on bile acids and the gut microbiome in CDI subjects have largely focused on the effects of FMT. Comparing stool bile acids from subjects with recurrent CDI before and after FMT, Weingarden et al. (51) found increases in DCA and LCA and decreases in CA, TCA, and chenodeoxycholic acid (CDCA). Whereas the study did not explicitly draw correlations between bile acids and bacterial families, their reported trends suggest positive associations between secondary bile acids and Bacteroidaceae, Lachnospiraceae, Rikenellaceae, and Ruminococcaceae. Similar results were reported by Seekatz et al. (34), who used regression analysis to find that OTUs in Lachnospiraceae, Ruminococcaceae, and unclassified Clostridiales were positively and negatively correlated with secondary and primary bile acids, respectively. The study also found a negative correlation between LCA and several OTUs classified as Proteobacteria. Allegretti et al. (2) noted that *Enterobacteriaceae*, a family belonging to proteobacteria, is a significant predictor of recurrent CDI (rCDI). The same study identified the ratio of DCA to GDCA as the most significant bile acid predictor for being in the rCDI cohort compared with those suffering from their first episode of CDI or healthy controls. Taken together, these previous studies consistently associate expansion of several Firmicutes families and concomitant reduction of Enterobacteriaceae post-FMT with restoration of secondary bile acids and resolution of the recurrence cycle.

The above associations are also detected by our rank correlation analysis, which examined the effects of different antibiotic therapies rather than FMT. This suggests that the correlations we detected are likely driven by the distribution of bile acid-metabolizing enzymes among commensal gut bacteria and that the secondary bile acid profile differences between ridinilazole and vancomycin subjects are primarily due to their differential impact on the intestinal bacterial populations. This is further supported by the novel association we identified between bile acids and Coriobacteriaceae, which correlated positively with secondary bile acids and negatively with primary bile acids. This family includes *Eggerthella lenta*, which are significantly more sensitive to vancomycin (MIC_90_ 2 μg/mL) than to ridinilazole (MIC_90_ > 512 μg/mL) (16). Strains of *E. lenta* have been shown to possess enzymes that can oxidize and epimerize bile acid hydroxyl groups to produce secondary bile acids (17).

There is weaker agreement across different studies on the positive associations between primary bile acids and specific bacterial groups. Brown et al. (8) reported a positive correlation between primary bile acids and *Enterococcus*, *Lactobacillus*, and *Streptococcus*. Another study on FMT by Mullish et al. (29) found the abundance of *Lactobacillus* to correlate positively with conjugated primary bile acids. In our study, we found only one family, *Enterobacteriaceae*, which significantly correlated positively with both conjugated and unconjugated primary bile acids. In comparison, we detected many more negative correlations with both conjugated and unconjugated primary bile acids.

One possible explanation for these observations is that BSH activity is more broadly distributed across different phylogenic groups, whereas the 7α-dehydroxylating enzymes have been well documented in only a handful of species within the Lachnospiraceae (*C. scindens, C hylemonae*) (36), Clostridiaceae (*C. sordellii*) (12), Peptostreptococcaceae (*C. hiranonis*) (23), and Coriobacteriaceae (*Eggerthella lenta*) (17) families. In this light, the positive association between secondary bile acids and other families such as Bacteroidaceae could reflect their BSH activity, which is a gateway enzyme upstream of 7α-dehydroxylation in the production of secondary bile acids. We did not detect any significant correlations between Lachnospiraceae and conjugated bile acids, suggesting that this family harbors minimal BSH activity. Similarly, very few human gut bacteria in *Enterobacteriaceae* harbor BSH (37), and thus their positive correlation with primary bile acids likely reflects an indirect association driven by the expansion of Proteobacteria in dysbiotic CDI subjects (28).

Alternatively, the association between bacterial families and secondary bile acids could be explained by “cross-feeding.” Well-known examples of cross-feeding by gut bacteria include the metabolism of complex plant oligosaccharides and host-derived mucins and the production of B vitamins and short-chain fatty acids. An in silico analysis of secondary bile acid metabolism by Heinken et al. (18) found that many gut bacteria possess genes for a part of the 7α-dehydroxylation pathway, although only a few species encode the complete pathway. Simulations performed in this study identified pairs of bacteria that could collaboratively synthesize secondary bile acids from conjugated primary bile acids. These pairs belong to Clostridiaceae, Ruminococcaceae, Lachnospiraceae, and Bacteroidaceae, families that our correlation analysis positively associated with secondary bile acids. The existence of collaborative pathways for bile acid metabolism spanning multiple species may explain the preservation of secondary bile acids in subjects treated with ridinilazole, which is active against *C. scindens* and *C. hylemonae*, in addition to *C. difficile* (15). Alternatively, there may be as-yet unsequenced ridinilazole-resistant species capable of converting primary bile acids and/or their conjugated salts into secondary bile acids. Potentially, both mechanisms may be active.

By comprehensively profiling representative bile acids from all four major categories, we were able to extend on previous studies to take a step toward delineating direct (enzymatic) and indirect associations between various bile acids and commensal gut bacteria. Our study is unique in the broad range of conditions under which samples were taken (healthy subjects and CDI patients, multiple time points over the course of antibiotic treatment), which enabled the discovery of a large number of significant associations, confirming in vivo metabolic capabilities that have been observed previously in vitro or in silico.

We did not quantify noncanonical, unsaturated bile acids such as cholenic acid, recently shown to be depleted in patients with CDI compared with patients with non-CDI related diarrhea (32). Although their role in CDI is unclear, it has been hypothesized that these bile acids may indicate an “extended host-microbiome dihydroxylation network.” Further studies are warranted to determine whether noncanonical bile acids are associated with particular taxa that are depleted in CDI and/or rCDI and, if so, whether these bile acids rebound following treatment, depending on treatment choice.

Despite one small case series that has shown reduced CDI relapse rates after administration of the secondary bile acid ursodeoxycholate compared with estimated historical control rates (50), preservation of bile acid-metabolizing capacity is unlikely to be the sole determinant of sustained clinical response, and other metabolites (20) are likely also important in restoring resistance to infection. However, restoration of normal bile acid homeostasis does appear to be a proxy marker for successful FMT. The number of subjects who tested positive for recurrence of CDI in the phase 2 clinical trial for which we had samples is relatively small (6 of 22 and 1 of 22 in the vancomycin and ridinilazole groups, respectively). Although we detected a statistically significant difference between recurrence and nonrecurrence cases, the small sample size limits the statistical power. Further studies are warranted to determine whether bile acids and/or taurine, a product of bile salt deconjugation, at EOT could indicate an individual’s likelihood of having a sustained clinical response.

In conclusion, ridinilazole is a promising, targeted-spectrum CDI antimicrobial that minimally disrupts commensal colonic flora and the associated bile acid metabolome. This microbiota- and metabolome-preserving property of ridinilazole may explain its favorable sustained clinical response rates compared with vancomycin treatment in the phase 2 trial.

## GRANTS

This work was supported by the National Science Foundation (1337760 to K. Lee), National Cancer Institute (CA211839 to K. Lee), and Summit Therapeutics, Plc (Abingdon, UK) (sponsored research agreement to K. Lee and C. M. Thorpe). X. Qian received a fellowship from the National Center for Advancing Translational Sciences, National Institutes of Health (TL1TR002546). K. Yanagi received a fellowship from Tufts University.

## DISCLOSURES

A. V. Kane has received research funds from Summit Therapeutics, Plc, and a travel grant from Summit Therapeutics, Plc. D. R. Snydman has received research funds from Merck, Pfizer, and Summit Therapeutics, Plc, and has been an advisor to Merck and Summit Therapeutics, Plc. R. J. Vickers is an employee of and holds share options in Summit Therapeutics, Plc. K. Lee has received research funds from Summit Therapeutics, Plc. C. M. Thorpe has received research funds from Merck (formerly Cubist/Optimer), Actelion, and Summit Therapeutics, Plc., has been on a Summit Therapeutics, Plc, Advisory Board, and has received a travel grant from Summit Therapeutics, Plc.

## AUTHOR CONTRIBUTIONS

A.K., D.R.S., R.J.V., K.L., and C.M.T. conceived and designed research; K.Y. and A.K. performed experiments; X.Q., K.Y., A.K., N.A., M.L., and K.L. analyzed data; X.Q., K.Y., A.K., K.L., and C.M.T. interpreted results of experiments; X.Q., K.Y., and K.L. prepared figures; X.Q., K.Y., K.L., and C.M.T. drafted manuscript; X.Q., K.Y., A.K., K.L., and C.M.T. edited and revised manuscript; X.Q., K.Y., A.K., D.R.S., R.J.V., K.L., and C.M.T. approved final version of manuscript.
